# Mitochondrial Damage-Associated Molecular Patterns: From Inflammatory Signaling to Human Diseases

**DOI:** 10.3389/fimmu.2018.00832

**Published:** 2018-05-04

**Authors:** Serge Grazioli, Jérôme Pugin

**Affiliations:** ^1^Pediatric Intensive Care Unit, Department of Pediatrics, University Hospital of Geneva, University of Geneva, Geneva, Switzerland; ^2^Department of Microbiology and Molecular Medicine, Faculty of Medicine, University of Geneva, Geneva, Switzerland; ^3^Intensive Care Unit, Department of Anesthesiology, Faculty of Medicine, University Hospital of Geneva, University of Geneva, Geneva, Switzerland

**Keywords:** damage-associated molecular pattern, mitochondria, inflammation, pro-inflammatory cytokines, sterile inflammation, alarmins

## Abstract

Over the recent years, much has been unraveled about the pro-inflammatory properties of various mitochondrial molecules once they are leaving the mitochondrial compartment. On entering the cytoplasm or the extracellular space, mitochondrial DAMPs (also known as mitochondrial alarmins) can become pro-inflammatory and initiate innate and adaptive immune responses by activating cell surface and intracellular receptors. Current evidence indicates that uncontrolled and excessive release of mitochondrial DAMPs is associated with severity, has prognosis value in human diseases, and contributes to the dysregulated process observed in numerous inflammatory and autoimmune conditions, as well as in ischemic heart disease and cancer. Herein, we review that the expanding research field of mitochondrial DAMPs in innate immune responses and the current knowledge on the association between mitochondrial DAMPs and human diseases.

## Introduction

Innate immunity represents the first line of defense of the human body against infection. This system can detect pathogens through the recognition of conserved molecular motifs, called pathogen-associated molecular patterns (PAMPs). The bindings of these molecules to pattern recognition receptors (PRRs), such as toll-like receptors (TLRs) and NOD-like receptors (NLRs), activates the immune system and triggers a response against the pathogen. Interestingly, it has been shown in the recent years that the human body is using this same system to signal tissue damage in absence of infection in a theory developed by Matzinger in 2004 (1). Thus, in context of major cellular stress, the cells release intracellular molecules in the extracellular space, acting as a stress signal for the body and named damage-associated molecular patterns (DAMPs or alarmins). Similarly to PAMPs, DAMPs are recognized by PRRs and able to trigger an immune response in various cells such as macrophages, dendritic cells, and neutrophils (2). Prototypical DAMPs include DNA, high-mobility group box 1 (3), or heat shock proteins (4) and are implicated in a growing list of inflammatory diseases.

**Figure 1:**
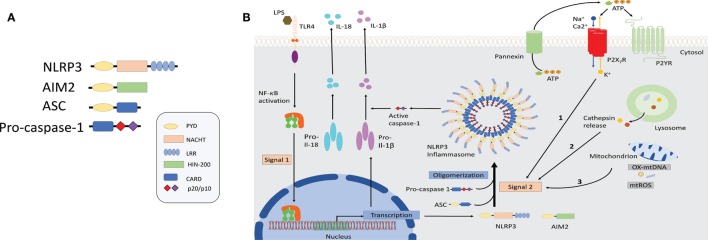
Schematic illustration of selected inflammasome components and mechanism of NLRP3 activation. **(A)** Shown here is the composition of NLRP3 and absent in melanoma-2 inflammasome sensors as well as the components ASC and caspase-1. **(B)** NLRP3 inflammasomes activation involves a two-step process: priming and assembly. Signal 1 (priming) is provided by nuclear factor-κB-dependent transcription of pro-interleuking-1β and NLRP3, either through the activation of TLRs or nucleotide-binding oligomerization domain 2 (NOD2) by microbial molecules or endogenous cytokines. The second signal is provided by stimuli that specifically activate NLRP3 and lead to NLRP3 oligomerization, caspase-1 activation followed by the maturation and release of IL-1β and IL-18. Three separate phenomena had been associated with NLRP3 activation. Event 1: ATP mediated ionic flux and intracellular potassium depletion mediated by ATP binding to ligand-gated ion channels P2X_7_R. Event 2: cathepsin release following destabilization of lysosomal membrane by sterile particulates, such as silica, asbestos, and cholesterol crystals. Event 3: generation of ROS and cytoplasmic release of mitochondrial DNA following major cellular stress and mitochondrial damage. Abbreviations: ATP, adenosine triphosphate; AIM2, absent in melanoma-2; ASC, apoptosis-associated speck-lick protein containing a CARD; CARD, caspase-activation and recruitment domain; HIN-200, hematopoietic interferon-inducible nuclear proteins with a 200-amino-acid repeat; IL-1β/IL-18, interleukin 1β/18; LPS, lipopolysaccharide; LRR, leucine-rich repeats; NBD/NACHT, nuclear binding domain; NF-κB, nuclear factor-κB; NLRP3, NLR family pyrin domain-containing; NLR, nucleotide-binding domain and leucine-rich repeat receptors; NOD2, nucleotide-binding oligomerization domain 2; PYD, pyrin domain; ROS, reactive oxygen species; TLRs, toll-like receptors.

It has recently been demonstrated that “danger” signals present in the cytoplasm in context of an active infection (PAMPs) or cellular damage (DAMPs) could also be detected by intracellular sensors of the innate immune system either directly or indirectly as a result of loss of homeostasis and trigger a pro-inflammatory immune response through the formation of inflammasomes (5, 6). Inflammasomes are multimeric protein complexes composed of a PRR, typically the NLR or the absent in melanoma 2 (AIM2) receptors that upon sensing its agonists oligomerizes and recruits the adaptor protein apoptosis-associated speck-like protein with a caspase-activation and recruitment domain (CARD) (ASC) to form a multimeric complex. Oligomerized ASC recruits pro-caspase-1 into the complex through CARD–CARD interaction, which then is converted into bioactive caspase-1 by autoproteolitic cleavage. Biologically active caspase-1 then processes the pro-inflammatory cytokines pro-IL-1β and pro-IL18 into their bioactive forms (Figure 1) (7,  8). For further details, the reader can refer to recent reviews in which all of the NLR inflammasomes have been discussed (9–11).

Recently, mitochondrial DAMPs have been identified as important mediators of the innate immune response and implicated in various conditions such as trauma, sepsis, and autoimmune disorders (Table 1). According to the endosymbiont theory of mitochondria, over a billion years ago, energy producing alpha bacteria were either engulfed by or invaded archezoan cells (12). Thus, the prokaryotic origin of the mitochondria could explain the numerous similarities they share with bacteria including a double membrane structure, an independent genome, which is circular in form, contains significant amount of unmethylated CpG motifs and encodes essential protein subunits of the oxidative phosphorylation system independently of nuclear DNA.

**Table 1:** Mitochondrial damage-associated molecular patterns.

mtDAMPs	Intracellular physiological role	Extracellular actions	Release pathways	Receptors	Implicated diseases
mtDNA	Coding for oxidative phosphorylation complex subunits (mitochondrial proteins)	Pro-inflammatory response; neutrophil activation (13, 14) and NET production (14); increase TLR9 expression in macrophages (15); endothelial cells activation (16) promoting neutrophils adhesion and transmigration	(a) Passive release (*via* necrosis, necroptosis (17, 18), apoptosis) (b) Active release [*via* mitochondria derived vesicles (19–22)]	TLR9, inflammasomes [NLRP3, AIM2 and NLRC4 (23–25)], STING (26–28)	Sepsis (29–37), trauma (31, 36, 38–43), cardiogenic shock (30, 44), cancer (45–52), liver failure (53, 54), heart failure (55), atherosclerosis (56), strokes (57), rheumatoid arthritis (58, 59), SLE (60–62)
ATP	Energy metabolism and coenzyme	Pro-inflammatory response; neutrophil chemotaxis, adhesion (63), phagocytosis (64, 65) and degranulation (66, 67); monocyte migration (68), adhesion (69), ROS production (23, 70, 71) and phagosome lysosome fusion (72); lymphocytes migration (73), activation (74) and proliferation (63); activation of NLRP3 inflammasome on monocytes (75, 76)	(a) Passive release by necrotic cells (b) Active secretion by vascular cells *via* vesicular exocytosis, and pannexin channel (77) (c) Active secretion by epithelial cells *via* CFTR dependent (78) or independent mechanism (79)	Purinergic receptors (P2X and P2Y)	Cancer (80, 81), asthma (82, 83), GVHD (84), lung diseases (85, 86), CF (87)
TFAM	Regulation of mtDNA transcription and stabilization	Pro-inflammatory response; synergistic effect with other mt DAMPs (N-formyl peptide and mtDNA) to increase cytokine production in monocytes (88) and dendritic cells (89); increase cytokines production in macrophages (90)	(a) Passive release *via* necrotic cells	Unknown	Heat failure (91), COPD (92)
N-formyl peptide (fMLP)	Share similarities with bacterial N-formyl peptide	Pro-inflammatory response; chemoattractant for neutrophils (93) and activates platelets (94)	(a) Passive release *via* necrotic cells	Formyl peptide receptors (FPRs)	Trauma with SIRS (95), liver injury (53), localized juvenile periodontitis (96)
Succinate	Intermediate synthetized in the TCA cycle or metabolite of cellular respiration	Pro-inflammatory response; triggers intracellular calcium mobilization, migration and has synergistic effect with TLRs ligands for proinflammatory cytokines production in dendritic cells (97); enhances antigen-specific activation of helper T lymphocytes; enhances IL-1β production in lipopolysaccharide-primed macrophages (98)	Unclear	GPR91 (97)	Pulmonary artery hypertension (99, 100)
Cardiolipin	Maintaining membrane potential and architecture and provides structural and functional support to protein involved in mitochondrial biogenesis	Pro-inflammatory response; activates inflammasome NLRP3 mediated immune response (101); activation and proliferation of gamma/delta T cells (102)		CD1d (102); NLRP3 (101), Atp8b1 (103)	Pneumonia (103), COPD (104)
Cytochrome-c	electron carrier in mitochondrial respiratory chain	Pro-inflammatory; cellular toxicity; induced lymphocytes (105) and neuronal cells (106) apoptosis	(a) Passive release via necrosis or apoptosis (107)	Apaf-1 (108)	Myocardial infarction (109), liver diseases (110, 111), cancer (112, 113), SIRS/MODS (114), acute encephalopathy (115), hemodialysis (116)

*mtDNA, mitochondrial DNA; NET, neutrophil extracellular trap; TLR9, toll-like receptor 9; NLRP3, NOD, LRR and pyrin domain-containing protein 3; AIM2, absent in melanoma 2; NLRC4, NLR family CARD domain containing 4; STING, stimulator of interferon genes; SLE, systemic lupus erythematosus; CFTR, cystic fibrosis transmembrane conductance regulator; ATP, adenosine triphosphate; TFAM, mitochondrial transcription factor A; COPD, chronic obstructive pulmonary disease; SIRS, systemic inflammatory response syndrome; MODS, multiple organ dysfunction syndrome; Apaf-1, apoptotic protease activating factor-1*.

Therefore, it is not really surprising that the release of mitochondrial molecules such as mitochondrial DNA (mtDNA) into the cytosol or the extracellular space triggers such a significant pro-inflammatory response.

To prevent the leak of pro-inflammatory mitochondrial molecules into the cytosol or the extracellular space in context of mitochondrial dysfunction, cells have developed various prevention systems. One of those systems is autophagy that enables cells to recycle damaged mitochondria and prevent leakage of its content into the cytosol or extracellular space.

However, in context of major cellular stress and tissue injury, the system may be overwhelmed, and mitochondrial DAMPS are released into the cytoplasm or the extracellular space, triggering a pro-inflammatory response.

There is a growing body of literature suggesting that mitochondrial DAMPs are implicated in various human diseases (117). Indeed, they are detected in different human body fluids suggesting its potential use as biomarkers for many diseases. The exact mechanism as to how mtDNA is released into the cytosol and the extracellular space is not completely characterized. In addition to a passive release of mtDNA in the context of cellular necrosis or apoptosis, some reports indicate that mitochondria DAMPs such as ATP or mtDNA can be actively secreted into the extracellular space. In these studies, it is suggested a more complex role of the mitochondria as an immunomodulatory of the innate immune system, and a potential therapeutical target for diseases characterized by an uncontrolled inflammation.

Herein, we review the nature and the role of the different mitochondrial DAMPs and discuss their interactions with different organs in the settings of sterile inflammation.

## Mitochondrial DAMPs: Function and Regulation

### Mitochondrial DNA

Mitochondrial DNA contains 37 genes coding for 2 ribosomal nucleic acids, 22 transfer RNAs, and 13 essential protein subunits of the oxidative phosphorylation system (118, 119). Specific characteristics of mtDNA are low methylation levels and heightened susceptibility to oxidative damage. To protect itself from oxidative damage, mtDNA is packaged into protein–DNA complexes called nucleoids under the control of the transcription factor A (TFAM). There are hundreds to thousands copies of mtDNA present in every cell of the human body with the exception of erythrocytes that lack mitochondria.

**Figure 2:**
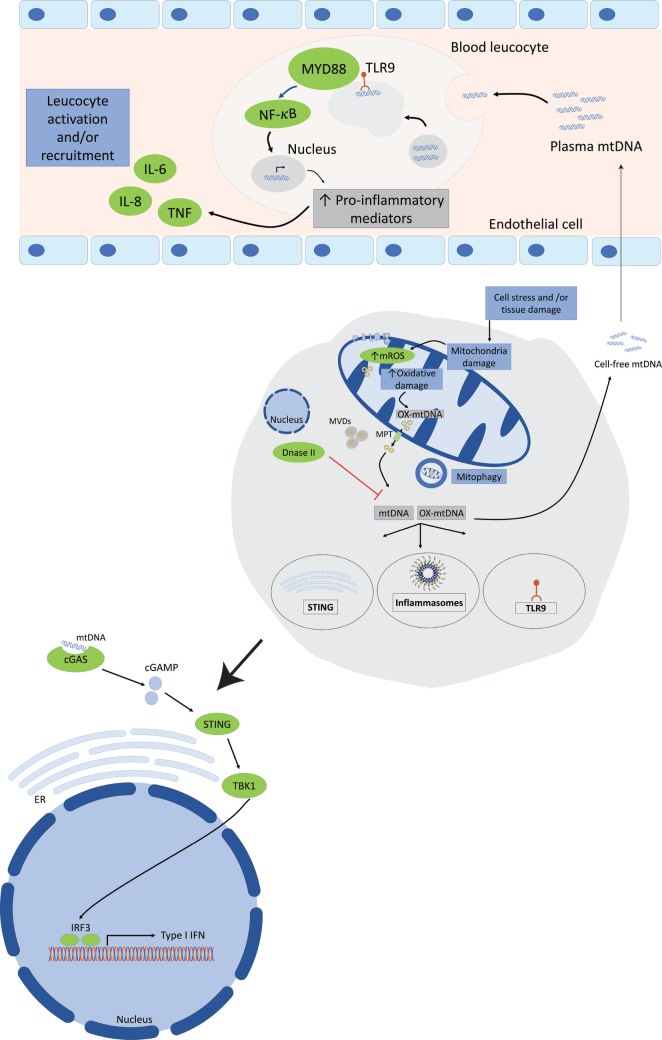
Mitochondrial DNA (mtDNA) mediates a pro-inflammatory response by interacting with TLR9, cytosolic inflammasomes, and type I interferon response. Tissue injury or cellular damage can cause mitochondrial dysfunction resulting in oxidative damage with increased mitochondrial reactive oxygen species (ROS) production and mtDNA oxidation. Oxidized mtDNA will be released into the cytosol and then to the extracellular milieu by various mechanisms including the transport in mitochondrial derived vesicles (MVDs) or through mitochondrial permeability transition pores (MPT). The whole damaged mitochondria can also be recycled through the mitophagy process. In the plasma, mtDNA activates TLR9-mediated signaling pathway in circulating neutrophils resulting in increased production of pro-inflammatory mediators, such as tumor necrosis factor (TNF), interleukin-6 (IL-6), and adhesion molecules. Cytosolic mtDNA can also engage and activate the NOD, leucine-rich repeats, and pyrin domain-containing protein 3 (NLRP3) inflammasome or absent in melanoma 2 inflammasome and trigger a pro-inflammatory response in cells primed with first signal in inflammasome activation through the nuclear factor (NF)-κB pathway. Finally, cytosolic mtDNA can also be recognized by and engage the cyclic GMP-AMP synthase (cGAS). Once activated cGAS triggers conformational changes of the endoplasmic reticulum-resident protein stimulator of interferon genes (STING), which engages TANK-binding kinase 1 to activate interferon regulatory factor 3 (IRF3) and/or IRF7 to stimulate transcription of type I interferons (IFNs) genes.

Mitochondrial DNA can be recognized by three important PRRs of the innate immune system, TLR9, cytosolic inflammasomes, and type I interferon response, and interactions of mtDNA with its receptors trigger a pro-inflammatory response (Figure 2).

#### MtDNA and TLR9

TLR9 is a member of the TLR family and is expressed mostly in immune cells including dendritic cells, macrophages, and naturel killer cells. TLR9 is widely referred as a DNA-sensing PRRs since it is able to detect hypomethylated CpG motifs of DNA present in both bacterial (120, 121) and viral DNA (122). Experimental studies on human macrophages and dendritic cells stimulated with labeled CpG DNA have demonstrated that CpG DNA is internalized into the lysosomal compartment followed by rapid translocation of TLR9 from its initial location in the endoplasmic reticulum to the CpG-rich lysosomal compartment for ligand binding and signal transduction (123). After the binding of TLR9 and its ligand, numerous downstream pathways will be activated, including pro-inflammatory nuclear factor kappa B (NF-κB), nucleotide-binding domain, and leucine-rich repeat (NLR) prying domain containing 3 (NLRP3) inflammasomes, and interferon regulatory factor-dependent type 1 IFN (124).

Due to its similarities with bacteria DNA, it is postulated that mtDNA can mediate a pro-inflammatory response dependent on the TLR9 pathway. This postulate was confirmed by substantial cellular and animal studies demonstrating capacity of mtDNA to activate the inflammatory/immune system *via* the TLR9 pathway. mtDNA injection in mice causes severe inflammatory response in the lungs (13, 125), which was reduced in *tlr9* −/− and *MyD88* −/− mice (125). In another study, using an animal model of acetaminophen-induced liver injury, oral acetaminophen administration to mice induced the release of mtDNA into the circulation and caused a marked systemic pro-inflammatory response with both liver injury and remote lung injury, which was prevented by TLR9 absence (*tlr9* −/− mice) (53). These animal studies confirmed that mtDNA activates innate immune response though TLR9 with an improvement of outcome when the TLR9 gene is deleted.

#### mtDNA and TLR9 p38 Mitogen-Activated Protein Kinases (MAPK) Pathway

Zhang et al. reported that mtDNA was released into the circulation during shock, both in humans and animal model of shock, activated human neutrophils p38 MAPK pathway, and triggered the release of MMP-8 and MMP-9 *via* TLR9 (38). More recently, Gu et al. (126) demonstrated that intratracheal instillation of mtDNA in mice activated p38 MAPK *via* TLR9 and caused significant lung inflammation with histological signs of acute lung injury accompanied by CD68^+^ macrophages infiltration.

#### mtDNA and Neutrophil Extracellular Traps (NETs)

Neutrophil extracellular traps are extracellular structure secreted by neutrophils that are composed of DNA and able to bind and kill microorganisms. Recent studies in trauma patients have shown that mtDNA was present in NETs and was able to induce NET formation in stimulated human neutrophils *via* TLR9 independently of NADPH oxidase (14).

#### mtDNA and Inflammasome

In addition of its role as DAMPs in the extracellular space, several reports have shown that mtDNA also mediates inflammatory responses in the intracellular space through inflammasomes activation. Several types of inflammasomes have been described with the NOD, leucine-rich repeats, and pyrin domain-containing protein 3 (NLRP3) and AM2 being the inflammasomes the most involved in mtDNA sensing.

#### mtDNA and NLRP3

The best characterized inflammasome, NLRP3, is composed of three key components: NLRP3, the ASC, and caspase-1 (Figure 1). For NLRP3 inflammasome to be assembled and activated, two signals are required: a priming signal that is necessary for the upregulation of both NLRP3 and pro-Il-1β transcription, often *via* NF-κB activation by a TLR or NOD2 agonist such as lipopolysaccharide (LPS), followed by an activation signal produced by crystals, aggregated β-amyloid, extracellular ATP, or microbial toxins that prompts NLRP3 to assemble the inflammasome complex (127, 128).

Nakahira et al. and Zhou et al. (23, 129) were the first to demonstrate a link between mitochondrial dysfunction and NLRP3 inflammasome activation. They showed that mtDNA mediates inflammatory response in the intracellular space though the activation of caspase-1 in response to LPS and ATP in bone marrow-derived macrophages (BMDM). Further, they demonstrated that mitochondrial ROS was required, upstream of mtDNA, for caspase-activation upon LPS and ATP treatment. Interestingly, they also found that NLRP3 inflammasome could increase cytosolic mtDNA release, which could play a role as a positive feedback loop, and further increase the pro-inflammatory response. Shimada et al. further characterized mtDNA and NLRP3 inflammasome interaction by showing that induction of mitochondrial damage in macrophages triggered an apoptotic signal that caused the cytosolic release of oxidized mtDNA that binds to and activated NLRP3 inflammasome (24).

#### mtDNA and AIM2

Absent in melanoma-2 represents another well described cytoplasmic DNA sensor. It belongs to the IFI20X-IFI16 (PYHIN) protein family and binds to DNA *via* hematopoietic interferon-inducible nuclear proteins with a 200-amino-acid repeat domain (130), whereas the pyrin domain associates with the adaptor molecules ASC to activate caspases (131, 132).

The groups of Nakahira et al. (23) and Shimada et al. (24) both showed that BMDM transfected with mtDNA and exposed to LPS and ATP had an increase in IL-1β production through a predominant AIM2 inflammasome activation. Recently, Yu et al. (133) were able to bring further clarification into the complex interaction between mitochondrial dysfunction, NLRP3 and the activation of AIM2 inflammasomes. They demonstrated that capsase-1-dependent mitochondrial damage represented a critical step in inflammasome activation. In addition, they showed that AIM2 inflammasome activation in BMDM causes caspase-1-dependent mitochondria damage and mitophagy inhibition, which represents cellular process to remove damaged mitochondria. Those studies confirmed that mitochondrial damage represented a critical step in inflammasome activation, with a potential role of mtDNA as direct ligand for NLRP3 and AIM2 inflammasome.

#### mtDNA and Type I Interferon Response

Recent reports have shown that mtDNA could also be recognized by another DNA sensing pathway of the innate immune system, namely, the cyclic GMP-AMP synthase (cGAS) and stimulator of interferon genes (STING) axis (26, 27) that trigger a type I interferon (IFN) response. cGAS–STING signaling axis represents an important regulator of type I IFN response of both exogenous and endogenous DNA (134–136). cGAS is a cytosolic DNA sensor that activates innate immune response through production of the second messenger cGAMP, which will activate the adaptor STING and induce type I IFN expression (IFN-α and IFN-β). After secretion, type I IFNs will bind to the type I IFN receptor (IFNAR), which will induce the expression of hundreds of interferon-stimulated genes providing a cellular state of viral resistance (137). The groups of Rongvaux et al. (26) and White et al. (27) have both demonstrated that Bax- and Bak-mediated mitochondrial apoptosis caused the release of mtDNA into the cytosol, which is recognized by the cGAS/STING pathway, and trigger a pro-inflammatory response with type I IFN induction in the absence of the apoptotic caspases.

These results suggest that the apoptotic cascade play a critical role to render mitochondrial apoptosis immunologically silent by suppressing DAMP signaling, such as mtDNA.

#### Mechanism for mtDNA Release to the Cytosol and Extracellular Compartments

Various mechanisms had been described to explain mtDNA release from the mitochondrial compartment into the cytosol. Several studies demonstrated the transfer of mtDNA from to the cytosol *via* mitochondrial membrane permeability transition (MPT). One of the first description of the MPT dates from the late 1970s when Haworth and Hunter showed in a series of publications (138–140) that Ca^2+^ uptake stimulated significant changes in mitochondrial morphology and functional activity mediated by the opening of a non-specific pore in the inner mitochondrial membrane. It is speculated that MPT are constituted of the association of several proteins located at contact sites between the inner and outer mitochondrial membranes (OMMs). Patrushev et al. (141) confirmed those results by demonstrating that MPT induction with exposition to inorganic phosphate trigger the release of mtDNA fragments without disturbing mitochondrial membrane integrity, in a process that was suppressed by cyclosporine treatment, an inhibitor of pore opening. Studies have shown that mitochondrial ROS were involved and could induce MPT. Interestingly, Nakahira et al. (23) have found in their study on mtDNA-induced NLPR3 inflammasome activation, that co-treatment with cyclosporine A was able to inhibit the secretion of IL-1β by macrophages exposed to LPS and ATP suggesting that the cytosolic release of mtDNA in their model was through increased MPT. In a recent publication, West et al. (28) demonstrated that an alteration in the mtDNA packaging system in the mitochondria could cause mtDNA to escape into the cytosol and be detected by the cGAS–STING signaling axis.

A significant side effect of mitochondrial electron transport is the generation of ROS that may oxidize mitochondrial protein with the risk to jeopardize the integrity and the function of the mitochondria. Recent studies have shown that mitochondria are able to remove oxidized proteins and lipids through small size vesicles, named mitochondria-derived vesicles (MDVs) targeting either peroxisomes (142) or lysosomes (143–146). Interestingly, they demonstrated that MDV delivery to the lysosomes requires PTEN-induced putative kinase-1 (pink1) and parkin in a pathway that was independent of the autophagy (mitophagy) machinery and did not require mitochondrial depolarization (144, 145). Since pink1 and parkin play a major role in the mitophagy process, those results suggest that the two proteins may also be involved in the removal of selected, oxidized cargo, in a process that run in parallel and maybe at an earlier time point than mitophagy. Whether oxidized mtDNA are transported in MDVs and how this new type of mitochondrial quality control mechanism prevents mtDNA-induced pro-inflammatory response remains unclear.

Although cellular necrosis with plasma membrane rupture constitutes a well described mechanism for the extracellular release of DAMPs, including mtDNA, some recent studies have shown that mtDNA could be released by cells undergoing a new described form of cellular death called necroptosis (17, 147, 148). Necroptosis represents a form of programmed necrosis that can be induced *via* death receptors, such as Fas or tumor necrosis factor-alpha (TNF) receptor leading to the activation of the receptor-interacting protein kinase 1 or 3 (RIP1/RIP3) (149–151). In an *in vitro* model of necroptosis, Maeda et al. (147) demonstrated that intact mitochondria were released by Jurkat cells and murine fibroblasts after TNF-α induced necroptosis before the disruption of the plasma membrane and could be sensed and phagocytosed by human macrophages, triggering the release of pro-inflammatory cytokines. Further supporting the active extracellular release of mitochondria and mitochondria components, experimental studies have shown that mitochondria could be actively transferred from bone-marrow-derived stroma cells to alveolar epithelial cells (19) and induce protection against acute lung injury or rescue aerobic respiration (20). Interestingly, Boudreau et al. (152) found that activated platelets secrete functional intact mitochondria into the extracellular space as free organelles or incorporated into microparticles. They showed that released mitochondria could be hydrolyzed by phospholipase A2-IIA-releasing mitochondrial DAMPs, including mtDNA, thus promoting leukocytes activation.

In a recent study, Caielli et al. brought some insights on the mechanism regulating mtDNA extracellular release in neutrophils (60). First, they demonstrated that neutrophils are spontaneously releasing mtDNA in the extracellular milieu in absence of cell death or membrane disruption, in a process enhanced by TLR7 engagement. Second, they showed that unlike other immune cells, neutrophils treated with CCCP to depolarize mitochondrial membrane are unable to complete the mitophagy process, suggesting that mtDNA extrusion may represent an alternative for the cell to remove oxidized mtDNA produced by damaged mitochondria. In addition, they observed that, in unstimulated neutrophils, oxidized mtDNA dissociated from TFAM and was exported into the cytosol in MVDs for lysosomal degradation. However, once the neutrophils are activated with IFN/αRNP, the dissociation of mtDNA-TFAM does not occur, impairing the clearance of oxidized mtDNA, resulting in the extracellular release of mtDNA still bond to TFAM.

#### Removal of mtDNA From the Cells

To prevent the leakage of the highly pro-inflammatory mtDNA both into the cytosol and the extracellular milieu, various cellular mechanisms have been described for the removal of mtDNA and damaged mitochondria. Autophagy, already mentioned in this review, represents a well described cellular mechanism for the removal of damaged organelles and is defined as mitophagy for the specific removal of damaged mitochondria. Numerous studies have demonstrated that blockade of autophagy results in the accumulation of dysfunctional mitochondria in the cells with increase mitochondrial ROS production and cytosolic release of mtDNA with NLRP3 activation (23, 153).

DNase II is an acid DNAse found in lysosome that plays an essential role in the degradation of DNA in apoptotic cells by macrophages after phagocytosis (154). Studies have shown that DNAse II plays a major role in mtDNA degradation during autophagy (55, 154). In an animal model of pressure overloaded-induced heart failure, Oka et al. (55) demonstrated that in mice lacking DNAse II, pressure overload resulted in mtDNA accumulation in autolysosomes leading to TLR9-induced pro-inflammatory response in cardiomyocytes.

### Mitochondrial RNA

Just like DNA, bacterial RNA constitutes other potent PAMP that is able to trigger an innate immune response. Numerous studies have demonstrated that bacterial RNA can be detected by cell type-specific endosomal and cytoplasmic receptors (155–159). In both human and murine plasmacytoid dendritic cells, bacterial RNA induces type I IFN response mediated by TLR7 (159), while in human monocytes bacterial RNA is detected by TLR8 resulting in IL-6 and TNF secretion (160). Interestingly, it has been shown that all three types of bacterial RNA (messenger RNA, transfer RNA, and ribosomal RNAs) were able to activate the NLRP3 inflammasome in human macrophages resulting in caspase-1-mediated cleavage of pro-IL-1β and pro-IL-18 into their active form (161). In a recent publication (160), Krüger et al. have demonstrated that specific mitochondrial ribosomal RNA segments similarly to bacterial ribosomal RNA could be detected by TLR8 receptors of PBMCs inducing a pro-inflammatory response with Il-6 and TNF secretion. Thus, those recent findings reinforce the endosymbiotic theory of mitochondrial origin and highlight a new intracellular alarm signal that can interact with the innate immune system and trigger a pro-inflammatory response.

### Adenosine Triphosphate (ATP)

Adenosine triphosphate is a nucleotide that has the particularity to play two distinct roles in the intracellular and in the extracellular spaces. ATP constitutes the main source of energy for most cellular processes. It is produced in mitochondria through the activation of the glycolysis pathway and the tricarboxylic acid (TCA) cycle. When found in the extracellular space, ATP plays a key role in cell signaling, as well as in the regulation in renal blood flow, vascular endothelium, and inflammatory responses. Moreover, it has been demonstrated that ATP released by injured or dying cells served as a DAMP to alert the immune system of tissue damage binding to a P2 purinergic receptors. ATP release seems to play key roles in interglia afferent signaling (including pain), in the regulation of renal blood flow, vascular endothelium, and in inflammatory responses. When ATP is released into the extracellular space, it constitutes important signaling molecules that has been involved in numerous inflammatory disorders by binding to purinergic P2 receptors. P2 purinergic receptors have a widespread expression throughout different tissues and are subdivided into P2Y receptors (P2YR) that are G-protein-coupled receptors and P2X receptors (P2XR), which are ligand-gated ion channels (Figure 1).

#### ATP and P2Y2R Signaling

**Figure 3:**
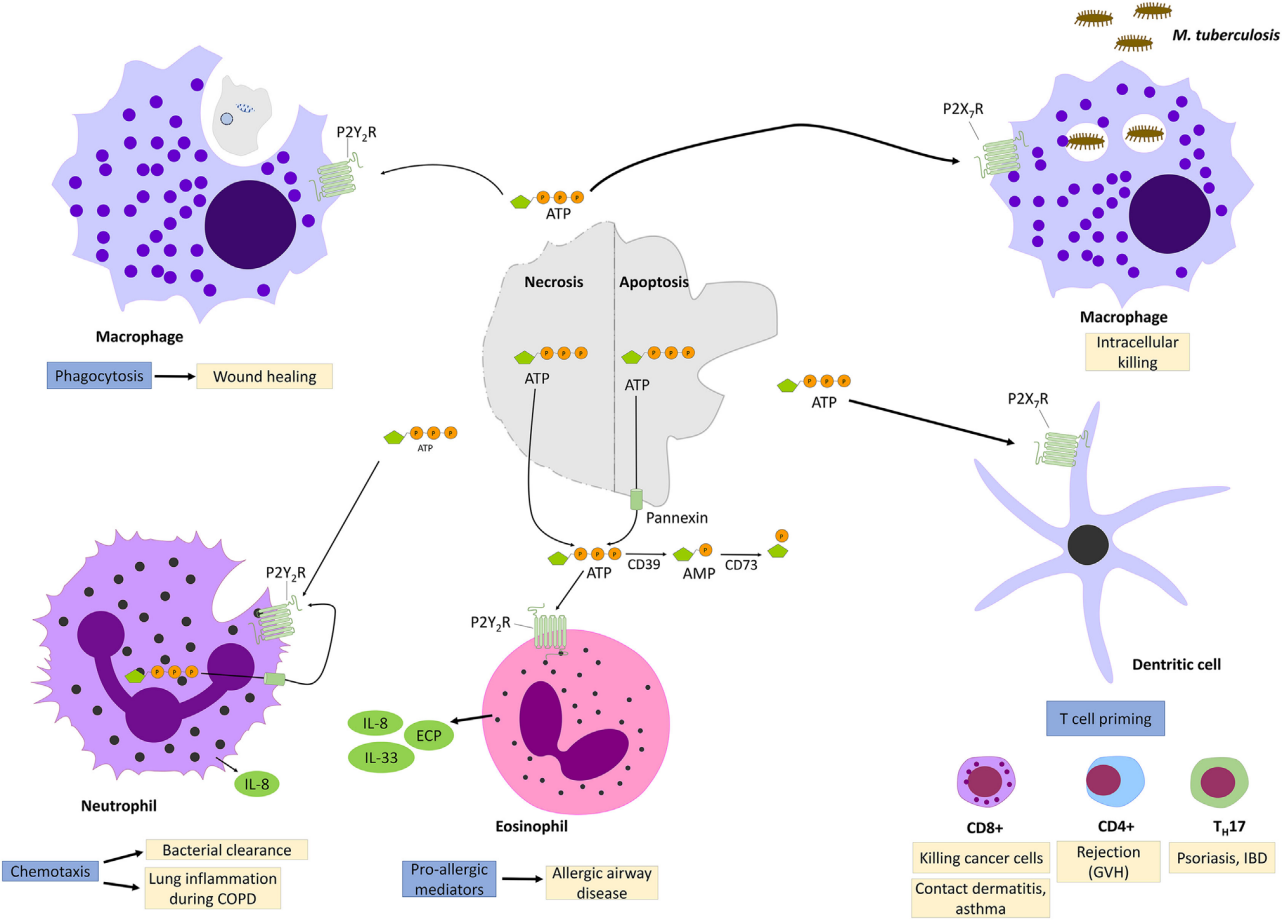
P2Y_2_R and P2X_7_R signaling during injury and inflammation. Adenosine triphosphate (ATP) can be released by necrotic cells or apoptotic cells though cytoplasmic channel, pannexin 1. When engaging P2Y_2_R, released ATP functions as “find me signal” for macrophages phagocytosis of injured cells and promotes so wound healing. P2Y_2_R signaling contributes also to bacterial clearance by stimulating neutrophils chemotaxis. However, excess P2Y_2_R signaling in neutrophils or eosinophils contributes, respectively, to chronic lung inflammation and allergic airway disease by promoting the release of pro-allergic mediators. P2X_7_R signaling promotes the killing of intracellular bacteria such as *Mycobacter Tuberculosis*. In dendritic cells, P2X_7_R signaling promotes T-cell priming which has been implicated in allergic diseases, graft-versus-host disease (GVH) as well as in psoriasis and inflammatory bowel disease.

Among all the P2YR receptors, P2Y_2_R signaling is the most studied and best characterized. A striking point that came out from all those studies is the complex and ambivalent effect that P2Y_2_R signaling can mediate, depending on the environment. On one hand, P2Y_2_R activation improves mucociliary clearance, bacterial clearance, and wound healing, but on the other hand, it contributes to a pro-inflammatory response in context of asthma et skin hypersensitivity (Figure 3).

Initially studied in cystic fibrosis patients because of the positive effect of P2Y_2_R agonists on mucociliary clearance mediated by the inhibition of the epithelial sodium channel ENaC (162), new studies reported the involvement of P2Y_2_R signaling in wound healing. It has been shown that ATP is released from apoptotic cells into the extracellular milieu through plasma membrane channel pannexin 1 and will act as a find-me signal that will bind to P2Y_2_R of macrophages and promote phagocytosis (163, 164). Further reports suggested a benefic effect of ATP in bacterial clearance based on neutrophil-dependent ATP release and autocrine activation of P2Y_2_R signaling (165). However, some other studies demonstrated that extracellular ATP contributes to a pro-inflammatory response through P2Y_2_R. It has been shown that P2Y_2_R stimulation with ATP induces the release of pro-allergic mediators such as IL-33, IL-8, and the eosinophil cationic protein (ECP) by human eosinophils and airway epithelial cells followed by a Th2-type innate immune response in mice exposed to allergens (82, 166).

#### ATP and P2X7 Signaling

P2X receptors are ligand-gated ion channels of which seven subtypes have been characterized (P2X1–7). Unlike P2YR, which can bind different ligands (ATP, ADP, UTP, UDP), P2XRs respond primarily to extracellular ATP. Binding to P2XR allow sodium and calcium influx and potassium efflux resulting in activations of p38 MAPK or phospholipase A2 mediated by the increased intracellular calcium level (167). Among all the P2XR subtypes, P2X7 receptors appears to be a key players in various inflammatory and infectious diseases and is capable of activating NLRP3 inflammasome (168) (Figure 3).

Indeed, several studies (72, 169, 170) demonstrated that ATP plays a major role in the innate immune response against intracellular bacterial infection such as tuberculosis or toxoplasmosis by activating P2X7 receptors in monocytes/macrophages, which results in a cascade of events promoting bacterial killing including ROS generation *via* NOX2 (70), improvement of phagosomes and lysosomes fusion (72) and apoptosis (169). Furthermore, loss of function of P2X7R was associated with increase susceptibility to tuberculosis or toxoplasma infection (170). In addition to its beneficial effect for the clearance of bacteria and parasites, it has been shown that ATP-P2X7 axis was also involved in tumor suppression (171). Several studies demonstrated that ATP was released from dying cancer cells and activated dendritic cells *via* P2X7 signaling, which then triggered NLRP3 inflammasome activation resulting in the secretion of IL-1β (171). IL-1β then primed IFN-gamma-producing CD8+ T cells, thus promoting the clearance of cancer cells.

Despite its important role in regulating appropriate inflammatory and immunological response against invasive pathogens or cancer cells, it becomes increasingly clear that inappropriate activation of ATP-P2X7 axis contribute to chronic inflammatory diseases.

Indeed, as already mentioned, P2X7R activation by extracellular ATP elicits NLRP3 inflammasome activation (172). Furthermore, it has been shown in various experimental studies using cellular model of allergic disorders (83) and inflammatory bowel disease (173) that ATP-mediated P2X7 signaling in dendritic cells resulted in T cell priming, and therefore, promoting a chronic inflammatory state.

### Mitochondrial Transcription Factor

Mitochondrial transcription factor A (TFAM), a member of the high mobility group box (HMGB) family, is essential for maintenance of mtDNA. It can bind to mtDNA in a sequence-independent manner and regulate both mitochondrial transcription initiation and mtDNA copy number (91, 174). Furthermore, it has been shown that TFAM constitutes an important regulator of mtDNA packaging into higher-order structures, namely nucleoids.

TFAM is stabilizing mtDNA through the formation of nucleoids. Several reports have demonstrated that TFAM can promote a pro-inflammatory response both intracellularly or when it is released into the extracellular space. West et al. (28) demonstrated that TFAM deficiency causes aberrant mtDNA packaging resulting in the cytosolic release of mtDNA, where it engages the DNA sensors sGAS and activates cGAS/STING pathway and IFN type I response. Interestingly, extracellular TFAM alone (90) or in presence of other DAMPs such as N-formyl peptide (88) or CpG DNA (89) can synergistically enhances inflammatory cytokines secretion in immune cells. Moreover, it has been shown that TFAM remains associated with mtDNA when it is released from injured cells and amplifies TNF-α release from TLR9-expressing dendritic cells by engaging RAGE (89).

### N-Formyl Peptide fMet-Leu-Phy

The N-formyl peptide (NFP) fMet-Leu-Phy is a very potent chemoattractant for immune cells (93, 175) and also acts on platelets (94). Chemoattractant formyl peptides are in the membrane of mitochondria and can be found in the extracellular milieu after cell membrane rupture or around dying cells. N-formyl peptides are also found in the membrane of bacteria. Once recognized by formyl peptide receptors (FPRs), a group of G protein-coupled chemoattractant receptors highly expressed on immune cells but also on platelets (94), NFP will participate in the clearance of damaged cells and host defense against bacterial infection (175–177). NFP can exert its chemoattractant effect on neutrophils either directly by binding to FPRs followed by an increase in intracellular Ca influx (175) or indirectly by inducing IL-8 secretion, another potent chemoattractant for neutrophils (88). Interestingly, co-stimulation with NFP and mtDNA has a synergistic effect on IL-8 secretion by neutrophils (13).

### Succinate

Succinate is a metabolic intermediate generated in mitochondria *via* the TCA cycle that plays an essential role in mitochondria function. Recent studies suggest that succinate, in addition of its crucial role ATP production, can also act as DAMPs and induce inflammation (98).

Once released in the extracellular environment, succinate can bind to the G protein-coupled receptor GPR91 (also named SUCNR1) that is highly expressed on the surface of dendritic cells (97) as well as in mouse kidney, liver, spleen, and small intestine (178). GPR91 signaling cascade has not been fully elucidated yet, but studies on human embryonic kidney 293 cells and human monocyte-derived dendritic cells have shown that GPR91 stimulation by succinate results in inositol triphosphate (IP3) accumulation, calcium mobilization, and extracellular signal-regulated kinase (ERK) phosphorylation (178). Dendritic cells treated with succinate displayed enhanced migration and inflammatory cytokines production through GPR91 signaling (97). Moreover, succinate synergizes with TLR ligands such as LPS to enhance the production of pro-inflammatory cytokines in dendritic cells. Interestingly, Tannahill et al. demonstrated in a recent publication that succinate was able to stabilize hypoxia-inducible factor-1 alpha (HIN-α), promoting inflammation in macrophages. Indeed, LPS-primed macrophages changed their core metabolism from oxidative phosphorylation to glycolysis which increased succinate level and IL-1β secretion mediated by stabilization of HIN-α by succinate (98). Inhibition of glycolysis suppressed LPS-induced IL-1β secretion but not TNF-α in mouse macrophage. Succinate was also reported to exacerbate inflammation by inducing the adaptive immune response. Indeed, succinate and antigen simultaneous exposition increased dendritic cells capacity for antigen-specific T cell activation (97).

### Cardiolipin (CL)

Cardiolipin is a lipid dimer consisting of two phosphatidyl group bridged by glycerol localized in the inner mitochondrial membrane that plays a critical role in diverse range of mitochondrial functions including mitochondrial respiration and mitochondrial biogenesis (179, 180). In addition to its role in maintaining membrane potential and architecture, CL is known to provide essential structural and functional support to several proteins involved in mitochondrial bioenergetics, including cytochrome oxidase and cytochrome bc1 (181, 182). Several reports have highlighted the close interaction between CL and cytochrome C. Indeed, ROS-mediated CL peroxidation causes the detachment of cytochrome C from the mitochondrial inner membrane and its release into the cytoplasm, where it induces apoptosis (183). Furthermore, it was reported that oxidized CL is translocated to the OMM, where it participates in formation of mitochondrial permeability transition pore, resulting in the cytosolic release of mitochondrial proapoptotic factors. Loss of CL or alterations in its acyl chain composition or CL peroxidation have all been associated with mitochondrial dysfunction present in human diseases such as ischemia, hypothyroidism, aging, and heart failure (180, 184). Several reports have suggested that cardiolipid may play a role in cell death. Indeed, it has been shown that during death receptor-mediated apoptosis, Fas-mediated, for example, CL moves from mitochondria to the cell surface where it is recognized by antiphospholipid antibodies (185, 186). Similarly to NFP and mtDNA, CL is present in bacteria, specifically in their cell wall, and is sequestered from the immune system in mammalian mitochondria. It was not such a surprise to show that CL can also induce inflammation by binding NLRP3 inflammasomes. Interference with CL synthesis has been shown to inhibit NLRP3 activation (101). To further support a role of cardiolipidin in the immune response, Dieude et al. demonstrated that CL can stimulate subset of CL-responsive γδ T cells present in the spleen and liver of healthy mice mediated by CL presentation by CD1d-bearing dendritic cells (102).

### Cytochrome-c

Cytochrome-c, a nuclear encoded, small soluble electron carrier located in the inner mitochondrial membrane, transfers electrons from complex III to IV (187, 188). Cytochrome-c is essential for the mitochondrial respiratory function, and its maintenance inside the mitochondria appears to be critical since its cytosolic release results in cell apoptosis. Once released into the cytosol, cytochrome-c binds and activates the apoptotic protease activating factor-1, which allows ATP binding and results in the formation of the ring-like apoptosome. Apoptosomes through CARD binds, and enzymatically activates procaspases 9, which will in turn activate effector caspase-3,-6, and -7, resulting in cell apotosis (108).

Numerous studies (189) have shown that cytochrome-c is released into the cytosol and then the extracellular space after mitochondrial injury and represent a useful marker of tissue injury in a large variety of diseases. In addition of being a marker of tissue injury, it has been shown that extracellular cytochrome-c can display pro-inflammatory proprieties mediated by the activation of NF-κB and causing neutrophil and monocyte triggered inflammation (190).

## Mitochondrial DAMPs in Human Diseases

### Mitochondrial DNA

#### mtDNA a Biological Marker of Tissue Injury

There is a growing body of literature demonstrating that circulating mtDNA levels are elevated in large variety of human diseases, including sepsis (29–36, 191, 192), trauma, pulmonary embolism (193), liver failure (53, 54), stroke (57), and cancer (45–50). Numerous recent clinical studies have suggested that mtDNA circulating level may represent a new biological marker of the severity of tissue injury for critical ill patient admitted with sever sepsis (29–36, 191, 192), trauma (13, 31, 36, 39–42), myocardial infarction (194, 195), and cardiogenic shock (44) since a correlation between mtDNA levels, injury severity and outcome has frequently been observed (30, 32, 36, 39, 41, 192). Interestingly, Krychtiuk et al. (30) demonstrated not only that mtDNA levels were associated with mortality in critical ill patients but also that TLR9 expression in patients increase the association between mtDNA and mortality. Furthermore, it was demonstrated in a clinical study with trauma patients that mtDNA levels represented an independent predictor for the development of post-traumatic systemic inflammatory response (SIRS) (40).

#### mtDNA in Chronic Inflammatory Disorders

Increased mtDNA levels were also detected in plasma and body fluid of patients presenting with a chronic inflammatory disorder (58, 59, 61). In patients with rheumatoid arthritis (RA) (58, 59), mtDNA increased in plasma and synovial fluid compared to healthy volunteers. Studies in animal model of RA demonstrated that both mtDNA intraarticular injection (59) or blockade of DNA degradation by using DNAse II deficient mice (59, 154) were associated with synovial cells activation and the development of chronic polyarthritis resembling to that of human RA. Together, these studies indicate that mtDNA is capable of inducing arthritis *in vivo* and may exacerbate inflammation by stimulating the release of pro-inflammatory cytokines, thereby creating a vicious cycle of sterile inflammation and cell destruction with further release of immunogenic mtDNA.

Although innate immunity plays a major role in the pathogenesis of systemic lupus erythematosus (SLE), recent reports have suggested that dysregulation of intra- and extracellular DNA degradation may be involved in human SLE (196, 197). Indeed, Caielli et al. were able to replicate their *in vitro* results by showing that not only neutrophils isolated from patient with SLE had an increase in oxidized mtDNA aggregates in their mitochondria compared to neutrophils from healthy patients or juvenile dermatomyositis patients but also that oxidized mtDNA represented an SLE autoantigen since they detected anti-oxidized mtDNA autoantibodies in SLE sera (60). Further supporting the role of mtDNA/anti-mtDNA antibodies in SLE, Wang et al. (62) detected the presence of mtDNA in NET secreted by neutrophils as well as anti-mtDNA antibodies in sera from SLE patients that correlated with the degree of IFN score and severity of nephritis. Altogether, these studies point to new biomarkers along with the classic anti-dsDNA autoantibodies that may correlate with patient degree of lupus nephritis as well as IFN inflammatory response.

### Adenosine Triphosphate

#### ATP and Cancer

Numerous studies have highlighted the importance of the immune response for the success of antineoplasic chemotherapies. In line with those evidences, recent studies have demonstrated that ATP released by dying cancer cell followed dendritic cell and inflammasome activation *via* P2X7R were critical steps for the success of the treatment of certain types of cancers (80, 81). Loss of function mutation of P2X7R in patients with chronic lymphatic leukemia (198) or breast cancer (171) are for example associated with increased risks of progression to metastatic disease state.

#### ATP and Asthma and Allergic Reaction

Human studies in patients with asthma suggest that ATP and purinergic signaling may contribute to the chronic inflammatory state of asthma. Analysis of bronchoalveolar lavage fluid in chronic asthma patients revealed increased extracellular ATP level (83) as well as upregulated P2X7R expression in airway macrophages (199) compared to healthy volunteers. In addition, human eosinophils isolated from asthmatic patients had also an increased P2X7 expression and released pro-allergic cytokines such as ECP and IL-8 in response to ATP simulation (166).

#### ATP and Inflammatory Disorders

A possible link between purinergic signaling and pathogenesis of psoriasis, a chronic skin disorder characterized by a Th17-dependent autoimmune response has recently been suggested (200). In this study, an upregulation of P2X7R in nonlesional and lesional psoriatic skin was observed compared with skin from healthy volunteers. In addition, they could elicit a proinflammatory response *via* P2X7 signaling by stimulating nonlesional psoriatic skin with ATP. Further studies have shown that purinergic signaling was also involved in inflammatory bowel diseases. Histological analyses of patients with Crohn’s disease have implicated the mast cells as the major effectors of the chronic intestinal inflammatory state. Recently, it was shown on colonic specimens of patients with Crohn’s disease, that P2X7R was upregulated in mast cells and that P2X7 receptor blockade or gene deletion in mice decreased intestinal inflammation (173). ATP and purinergic signaling have also been implicated in lung inflammatory disorders. In a mice model of bleomycin-induced lung injury, ATP levels were increased in bronchoalveolar lavage fluid and intranasal ATP injection further enhanced bleomycin-induced lung inflammation that could be inhibited with apyrosis treatment or in P2X7R knockout mice (85). Furthermore, it was shown that chronic obstructive pulmonary disease (COPD) patients had elevated ATP level in their lung lavage fluid with highest ATP concentrations among heavy smokers compared to non-smokers COPD patients. In addition, they demonstrated that ATP levels correlated negatively with lung function and positively with bronchoalveolar lavage fluid neutrophils, suggesting that airway ATP may represent a biomarker for smoke induced lung injury.

### N-Formyl Peptide, Formyl-Met-Leu-Phe (fMLP)

A recent study suggested that fMLP released in context of trauma could be responsible for the respiratory failure associated with trauma. In this study, Wenceslau et al. measured an increase in fMLP levels in plasma from trauma patients with SIRS or sepsis compared to control trauma group (95). In addition, they observed that fMLP induced lung neutrophil infiltration when instilled intratracheally to rats and produced airway contraction mediated by the fMLP receptor (FPR) and mast cells when exposed to isolated airways. The importance of fMLP and its receptor FPR for appropriate chemotaxis and immune response against infection was demonstrated in patients with localized juvenile periodontitis (LJP) (96). Indeed, it was shown that neutrophils from LJP patients present a genetic alteration in the gene coding for FPR, which could explain the decreased chemotactic activity and higher susceptibility for infections that is reported in LJP patients.

### Succinate

Extensive evidence from the literature suggests that mitochondria play an important role in the pathophysiology of pulmonary hypertension (PH) with mitochondrial alteration being observed in pulmonary arteries and in the right ventricle in context of PH associated with a metabolic shift from mitochondrial oxidative phosphorylation to glycolysis (99). Interestingly, it was shown in a recent study using an animal model of PH that pulmonary artery banding increased GPR91 and p-Akt expression in cardiomyocytes and that succinate treatment further increased p-Akt levels and right ventricle hypertrophy (99, 100). Those results suggest that succinate-GPR91 signaling is involved in right ventricle hypertrophy secondary to PH.

### Cardiolipin

Under normal conditions CL constitutes only 1–2% of alveolar surfactant but its level increases in animal model of lung injury (201, 202). The role played by surfactant composition and CL during lung injury was further underlined in a study from Ray et al. (103). Indeed, they demonstrated that CL levels were increased in bronchoalveolar lavage fluids from humans and mice with bacterial pneumonia. In addition, intratracheal instillation of CL in mice reproduces the clinical picture of pneumonia and impairs surface tension-lowering activity of the surfactant. Interestingly, this group identified and characterized the activity of a novel CL transporter, Atp8b1 that may play a role in the removal of CL from lung fluid. Mutation in *Atp8b1* was associated with severe pneumonia in humans and mice.

### Cytochrome-c

Extracellular release of cytochrome-c had been confirmed in various conditions characterized by cell death. Indeed, increased serum cytochrome-c level was reported in patients with myocardial infarction (109) as well as in patients with liver diseases (110,  111). In the oncology literature, cytochrome-c was reported both as prognostic markers in various cancers (112) and as a marker of apoptosis to monitor the efficiency of chemotherapy-induced cell death (113). Similar to mtDNA, cytochrome-c level are elevated in plasma of patients with SIRS compared to healthy volunteers and are associated with survival and organ dysfunction (114). Further supporting a possible role of cytochrome-c as a biomarker, it was shown that cytochrome-c levels were increased in patients with severe encephalopathy (115) as well as in patients on hemodialysis (116) and correlated, respectively, with patient’s outcome and IL-6 levels.

### Concluding Remarks

In this review, we discussed the function and molecular regulation of various mitochondrial DAMPs and their role in human diseases. This review highlighted the complexity of the interaction between mitochondria, inflammation, and the immune system. It has become increasingly evident that mitochondrial DAMPs play critical role in sterile inflammation and in various diseases such as those characterized by chronic inflammation. However, many questions remain unanswered. These include the mechanisms for mtDNA release to the outside of the cells and the origin of cell free circulating mtDNA, particularly in critical ill patients. Although, an increasing number of studies have demonstrated that mitochondrial DAMPs can be actively released by non-necrotic cells exposed to an external stimulation, the mechanism for extracellular release for example *via* MDVs or autophagy requires further clarification. Also, while our understanding of the function and regulation of each individual mitochondrial DAMPs is constantly improving, additional studies are warranted to clarify the biological relevance of the interactions between different mitochondrial DAMPs and other extracellular molecules. The interplay between the various DAMPs is also a key to mediate sterile inflammation. Although not discussed herein, DAMPs also frequently synergize with pathogen-associated molecular pattern.

Finally, therapeutic immunomodulation by targeting mitochondrial DAMPs and their signaling pathways is promising in various inflammatory and auto-immune diseases. The measurement of mitochondrial DAMPs in body fluids as a biological marker of disease may also be of utility and bear prognostic values.

## Author Contributions

SG wrote the manuscript, and JP edited and provided direction for the article.

## Conflict of Interest Statement

The authors declare that the research was conducted in the absence of any commercial or financial relationships that could be construed as a potential conflict of interest.
